# Ideal cardiovascular health and risk of death in a large Swedish cohort

**DOI:** 10.1186/s12889-024-17885-4

**Published:** 2024-02-02

**Authors:** Lijie Ding, Marta Ponzano, Alessandra Grotta, Hans-Olov Adami, Fuzhong Xue, Ylva Trolle Lagerros, Rino Bellocco, Weimin Ye

**Affiliations:** 1 Department of Medical Epidemiology and Biostatistics, Karolinska Institutet, Box 281, Stockholm, SE17177 Sweden; 2 Department of Health Management, Shandong Sports University, Jinan, China; 3 Department of Health Sciences, University of Genoa, Genoa, Italy; 4 Department of Statistics and Quantitative Methods, University of Milano, Bicocca, Italy; 5 Department of Public Health Sciences, Stockholm University, Stockholm, Sweden; 6 Centre for Health Equity Studies, Stockholm University/Karolinska Institutet, Stockholm, Sweden; 7 Clinical Effectiveness Group, Institute of Health and Society, University of Oslo, Oslo, Norway; 8 Department of Biostatistics, School of Public Health, Shandong University, Jinan, China; 9 Unit of Clinical Epidemiology, Department of Medicine, Karolinska Institutet, Stockholm, Sweden; 10 Center for Obesity, Academic Specialist Center, Stockholm Health Services, Stockholm, Sweden; 11 Department of Epidemiology and Health Statistics & Key Laboratory of Ministry of Education for Gastrointestinal Cancer, Fujian Medical University, Fuzhou, China

**Keywords:** Cardiovascular diseases, Ideal cardiovascular health, Cohort studies, Mortality, Sweden

## Abstract

**Background:**

Ideal cardiovascular health (CVH) can be assessed by 7 metrics: smoking, body mass index, physical activity, diet, hypertension, dyslipidemia and diabetes, proposed by the American Heart Association. We examined the association of ideal CVH metrics with risk of all-cause, CVD and non-CVD death in a large cohort.

**Methods:**

A total of 29,557 participants in the Swedish National March Cohort were included in this study. We ascertained 3,799 deaths during a median follow-up of 19 years. Cox regression models were used to estimate hazard ratios with 95% confidence intervals (95% CIs) of the association between CVH metrics with risk of death. Laplace regression was used to estimate 25th, 50th and 75th percentiles of age at death.

**Results:**

Compared with those having 6–7 ideal CVH metrics, participants with 0–2 ideal metrics had 107% (95% CI = 46-192%) excess risk of all-cause, 224% (95% CI = 72-509%) excess risk of CVD and 108% (31-231%) excess risk of non-CVD death. The median age at death among those with 6–7 vs. 0–2 ideal metrics was extended by 4.2 years for all-causes, 5.8 years for CVD and 2.9 years for non-CVD, respectively. The observed associations were stronger among females than males.

**Conclusions:**

The strong inverse association between number of ideal CVH metrics and risk of death supports the application of the proposed seven metrics for individual risk assessment and general health promotion.

**Supplementary Information:**

The online version contains supplementary material available at 10.1186/s12889-024-17885-4.

## Introduction

Ideal cardiovascular health (CVH), a concept developed by the American Heart Association (AHA) as part of its 2020 Strategic Impact Goal, aims at lowering the prevalence of risk factors to achieve primary prevention and reduced mortality [1]. The CVH defines ideal, intermediate and poor levels of four health behaviors (smoking, body mass index, physical activity, diet) and three clinical characteristics (blood pressure, total cholesterol, fasting glucose) [1, 2].

In several studies around the world, only a small proportion of subjects have ideal CVH [3, 4] with a higher prevalence in European countries than in the US [5]. An inverse association of ideal CVH metrics with both cardiovascular diseases (CVD) and all-cause mortality has been shown in US [6–8], UK [9], Chinese [10, 11] and Korean [12] populations with additional support from a meta-analysis [13]. However, neither the prevalence of ideal CVH, nor its association with risk of death has been reported in the Nordic countries. Moreover, an association of ideal CVH with CVD, cancer, and metabolic diseases like non-alcoholic fatty liver disease has also been identified [14–16], indicating that ideal CVH can be widely applied in the prevention of non-communicable diseases. In practice, tackling smoking, alcohol drinking, unhealthy diet, and physical inactivity as primary prevention strategy has been widely used, but the burden of cardiovascular diseases is still heavy. Thus addressing cardiovascular health in middle age has been advocated and lifelong prevention of chronic diseases is needed. In the promotion of ideal CVH for the primary prevention of chronic diseases, gain in life years by adopting healthy lifestyle could be a useful tool to show the positive aspects of avoiding risk factors [17, 18]. To this end, we aimed to explore the relationship of ideal CVH with all-cause, CVD and non-CVD death in a large Swedish cohort, by reporting life-years gained in addition to risk ratios.

## Materials and methods

### Study population

The detailed design of the Swedish National March Cohort has been reported previously [19, 20]. In brief, this study was established during a national fund-raising event for the Swedish Cancer Society in almost 3,600 Swedish cities and villages in September 1997. Participants were asked to fill out a 36-page questionnaire concerning socio-economic factors, lifestyle, dietary habits, anthropometric measures, and medical history. Participants provided their individually unique national registration numbers assigned to all Swedish residents, which allow follow-up through linkage to existing national registries [21].

In total 43,865 participants completed the questionnaire. We excluded participants with incorrect national registration numbers, conflicting answers (*n* = 11), age below 18 years (*n* = 1,740), death (*n* = 8) or emigration (*n* = 41) before start of follow-up, a history of myocardial infarction (*n* = 665), stroke (*n* = 440), heart failure (*n* = 116) or cancer (*n* = 2,667) at entry, or incomplete baseline information of any of the 7 ideal CVH metrics (*n* = 10,148). Thus, a total of 29,557 participants were included in the final analysis. The study was approved by the Regional Ethical Review Board at Karolinska Institutet and all study participants provided informed consent.

We obtained information on educational level, smoking habits, body mass index (BMI), physical activity, diet, diabetes, lipid disturbance, and hypertension by self-reported questionnaire. Education was assessed as the highest level attained, and classified into 3 categories: primary (≤ 9 years), secondary (10–12 years) and higher (> 12 years). BMI was calculated by dividing weight (kg) by squared height (m^2^). Physical activity was assessed by asking respondents about total time (hours/week) for light, strenuous and hard sports/exercise/outdoor activity during last 12 months [19]. Dietary information was obtained from an 85-item food frequency questionnaire (FFQ), a slightly abbreviated version of a validated 96-item FFQ questionnaire [22]. Participants reported how often they usually consumed each food and beverage item. Eight alternatives were available ranging from “0 times per month” to “3 or more times per day”. Finally, participants who self-reported ever being treated by a doctor for diabetes (yes/no), lipid disturbance (yes/no) and hypertension (yes/no) were classified as having these conditions.

### Definition of health metrics

In accordance with the AHA definition [1, 2], four CVH behaviors (smoking, BMI, physical activity, diet) were classified into ideal, intermediate and poor levels. BMI was categorized as ideal (< 25 kg/m^2^), intermediate (25 to 30 kg/m^2^) and poor (≥ 30 kg/m^2^). Physical activity was classified into ideal (≥ 150 min/wk moderate, or ≥ 75 min/wk vigorous, or ≥ 150 min/wk moderate + vigorous), intermediate (1–149 min/wk moderate, or 1–74 min/wk vigorous, or 1–149 min/wk moderate + vigorous if not reaching ideal level), and poor (none). For diet, there are 5 healthy diet components, which are ≥ 4.5 cups of fruits and vegetables per day, ≥ 2 servings (3.5-oz) of fish per week, ≥ 3 servings (1-oz equivalent) fiber-rich whole grains per day, < 1,500 mg sodium per day, ≤ 450 kcal (36 oz) sugar-sweetened beverages per week. Participants with 4–5, 2–3, 0–1 healthy diet components were categorized into ideal, intermediate and poor. For smoking, we defined participants as ideal (never-smokers), intermediate (ex-smokers), or poor (current smokers) according to their self-reported history of smoking, which differs from the AHA’s definition that ideal smoking was defined as never or quit > 12 months ago. For the other three CVH factors, participants who self-reported having diabetes, lipid disturbance, and high blood pressure at baseline were considered as poor level, and otherwise they were classified as ideal level. Since we were restricted to questionnaire data, we could not define ideal CVH exactly in the same way as the AHA’s definition, where diabetes and lipid disturbance are based on laboratory values and hypertension based on objectively measured blood pressure. Given the interest and complexity in alcohol and health [23], we also tried to include alcohol consumption in the model. Current Nordic Nutrition recommended thresholds are less than 10 g/day and 20 g/day respectively for men and women. We thus defined participants as ideal in terms of alcohol consumption based on these thresholds [24].

### Follow-up

Follow-up started on October 1, 1997 and continued until December 31, 2016, death or emigration from Sweden, whichever occurred first. Date of death was obtained from the Swedish Death Register and date of emigration from the Emigration Register [21]. Moreover, we identified CVD deaths from the Cause of Death Register (individuals with CVD (ICD-10 I00-I99) as the underlying cause of death).

### Statistical analysis

Continuous variables are presented as mean (standard deviation (SD)) and categorical variables are summarized as frequencies (percentages). To examine the relationship between the number of ideal CVH metrics and risk of death, we classified participants into five groups: <=2, 3, 4, 5 and 6–7 ideal CVH metrics at baseline. We used direct standardization method to calculate the age- (5-year band) and sex-standardized incidence of all-cause, CVD and non-CVD death with person-years distribution by age (5-year band) and sex categories in the total cohort as reference.

We used Cox proportional hazards regression models to estimate hazards ratio (HR) and 95% confidence interval (95% CI) for the association between the total number of ideal CVH metrics ( < = 2, 3, 4, 5 vs. 6–7) and all-cause, CVD and non-CVD death separately, after adjusting for age, sex and education, with time-on-study as the time-scale [25]. Linear trends of HRs were tested by using the grouping of ideal CVH metrics as a continuous variable in the Cox regression model. We also performed separate analyses, by dividing follow-up into the first 10 years and 10 + years. Since it is known that there exist some differences in the associations between males and females, we also performed the Cox regression analyses stratified by sex [26]. Likelihood ratio tests were used to assess interactions between age at baseline and the number of CVH metrics. Interaction terms were built considering age, both as a continuous and as a categorical variable (< 65, >=65 years). We also evaluated the presence of additive interaction between categorical age and categorical ideal health metrics ( < = 2 vs. 3+, <=3 vs. 4+, <=4 vs. 5+, <=5 vs. 6–7) using the relative excess risk due to interaction (RERI), while adjusting for education and sex. To control for potential bias due to reverse causality, we ran a sensitivity analysis by excluding the first two years of follow-up. Cox proportional hazards regression models were also used to estimate HRs for each single CVH metric (intermediate and poor vs. ideal) after adjusting for age, sex and educational level. As a sensitivity analysis all the CVH metrics were included in the Cox regression model and we also repeated the analyses by including individuals who had one missing out of the 7 ideal CVH metrics. Proportional-hazards (PH) assumption was tested using Schoenfeld residuals and the log-log plot of survival; we ran stratified Cox models for the covariates that did not satisfy the PH assumption. Specifically, the stratified Cox procedure allows to fit a Cox PH model when one or more of the explanatory variables do not satisfy the PH assumption [27]. Kaplan-Meier curves for the different categories of CVH metrics and also for different levels of each metric were plotted. Additionally, to assess the impact of the competing events, we replicated the main analyses using Fine-Gray models for CVD and non-CVD deaths.

Receiver Operating Characteristic (ROC) analysis was applied to evaluate the discriminatory capability of the number of ideal CVH metrics on risk of all-cause, CVD death and non-CVD death based on Cox model. To complement HR estimates, we also performed quantile regression analysis in order to assess associations of CVH metrics on gain of age in years. Specifically, we used Laplace regression to estimate 25th, 50th and 75th percentiles of age at death considering number of CVH metrics ( < = 2 vs. 3, 4, 5, 6–7) and each single CVH metric (intermediate and poor vs. ideal) as the main exposures, with age as the time scale and adjusting for age at baseline, sex and educational level [28]. We also performed Laplace regression separately for males and females.

Data analyses were performed with SAS statistical software version 9.4 (SAS Institute Inc, Cary, NC) and Stata version 15.1 (Stata Corporation, College Station, TX, USA).

## Results

### Baseline characteristics

Table 1 presents baseline characteristics and prevalence of single ideal CVH for the total cohort, as well as for different ideal CVH categories. Among 29,557 participants, 65.8% were women and the mean (SD) age at baseline was 48.6 (15.5) years (range 18.0-93.6, median 49.7). Among the four healthy behaviors, the prevalence of never smokers and ideal BMI was high overall. Ideal non-smoking, ideal BMI, and ideal physical activity were present in 64.2%, 62.2% and 34.9% of the total cohort. Concerning BMI, for 436 individuals (1.48%) the BMI was below 18.5 Kg/m^2^. However, only 11.8% met the ideal diet definition. The prevalence of self-reported diabetes, lipid disturbance, and high blood pressure was 1.9%, 2.7% and 10.2%. Overall, only 2.0% of the cohort participants had all 7 ideal CVH metrics, 20.3% had at least 6 ideal CVH metrics, and the majority had 4 or 5 ideal metrics (64.2%).

**Table 1:** Baseline characteristics of participants in the Swedish National March Cohort

	Total	Ideal health metrics, n				
	(*n* = 29,557)	0-1-2 (*n* = 839)	3 (*n* = 3740)	4 (*n* = 8739)	5 (*n* = 10,229)	6–7 (*n* = 6010)
Age, Mean (SD)	48.63(15.54)	59.31(10.38)	53.65(12.77)	50.65(14.08)	47.31(15.77)	43.33(17.03)
Sex, n (%)						
Male	10,118(34.23)	397(47.32)	1465(39.17)	2923(33.45)	3177(31.06)	2156(35.87)
Female	19,439(65.77)	442(52.68)	2275(60.83)	5816(66.55)	7052(68.94)	3854(64.13)
Education, n (%)						
Primary	9862(33.86)	426(51.51)	1638(44.38)	3312(38.48)	3106(30.82)	1380(23.30)
Secondary	10,034(34.45)	231(27.93)	1158(31.37)	2861(33.24)	3479(34.52)	2305(38.91)
Higher	9230(31.69)	170(20.56)	895(24.25)	2433(28.27)	3493(34.66)	2239(37.80)
BMI, n (%)						
Ideal	18,392(62.23)	62(7.39)	400(10.70)	3841(43.95)	8278(80.93)	5811(96.69)
Intermediate	9148(30.95)	550(65.55)	2637(70.51)	4076(46.64)	1699(16.61)	186(3.09)
Poor	2017(6.82)	227(27.06)	703(18.80)	822(9.41)	252(2.46)	13(0.22)
Smoking, n (%)						
Ideal	18,990(64.25)	133(15.85)	866(23.16)	4360(49.89)	7942(77.64)	5689(94.66)
Intermediate	8177(27.67)	611(72.82)	2254(60.27)	3249(37.18)	1797(17.57)	266(4.43)
Poor	2390(8.09)	95(11.32)	620(16.58)	1130(12.93)	490(4.79)	55(0.92)
Physical activity, n (%)						
Ideal	10,331(34.95)	19(2.26)	176(4.71)	1265(14.48)	3596(35.15)	5275(87.77)
Intermediate	14,561(49.26)	513(61.14)	2480(66.31)	5618(64.29)	5333(52.14)	617(10.27)
Poor	4665(15.78)	307(36.59)	1084(28.98)	1856(21.24)	1300(12.71)	118(1.96)
Diet, n (%)						
Ideal	3496(11.83)	22(2.62)	74(1.98)	414(4.74)	1072(10.48)	1914(31.85)
Intermediate	17,715(59.94)	612(72.94)	2588(69.20)	5652(64.68)	6201(60.62)	2662(44.29)
Poor	8346 (28.24)	205(24.43)	1078(28.82)	2673(30.59)	2956(28.90)	1434(23.86)
Diabetes, n (%)						
Ideal (no)	29,004(98.13)	628(74.85)	3570(95.45)	8626(98.71)	10,178(99.50)	6002(99.87)
Poor (yes)	553(1.87)	211(25.15)	170(4.55)	113(1.29)	51(0.50)	8(0.13)
Lipid disturbance, n (%)						
Ideal (no)	28,762(97.31)	536(63.89)	3509(93.82)	8542(97.75)	10,169(99.41)	6006(99.93)
Poor (yes)	795(2.69)	303(36.11)	231(6.18)	197(2.25)	60(0.59)	4(0.07)
High blood pressure, n (%)						
Ideal (no)	26,538(89.79)	128(15.26)	2625(70.19)	7908(90.49)	9910 (96.88)	5967(99.28)
Poor (yes)	3019(10.21)	711(84.74)	1115(29.81)	831 (9.51)	319(3.12)	43(0.72)

### Mortality by CVH category

During a median follow-up duration of 19.25 years, 3,799 deaths (1,261 CVD deaths and 2,538, non-CVD deaths) were observed and the crude incidence density of death was 7.05 per 1,000 person-years (3,799 /538,889), with 2.34 and 4.71 per 1,000 person-years for CVD and non-CVD death respectively. For 0–2, 3, 4, 5, 6–7 ideal metrics, the age-, sex-standardized incidence density (total cohort as reference) of death became 10.98, 8.24, 7.52, 6.32 and 5.55 per 1,000 person-years. Kaplan-Meier curves were plotted for the different categories of ideal metrics, as well as for different levels of each metric (Supplementary Fig. 1 and Fig. 2). Participants with 0–2, 3 or 4 ideal metrics had a significantly higher risk of all-cause, CVD and non-CVD death compared to those having at least 6 ideal metrics (Table 2). When we compared individuals with 0–2 ideal metrics to those with 6–7 ideal metrics, the HR for all-cause death increased by 107% (HR = 2.07; 95% CI = 1.46–2.92), the HR for CVD death increased by 224% (HR = 3.24; 95% CI = 1.72–6.09) and the HR for non-CVD death increased by 108% (HR = 2.08; 95% CI = 1.31–3.31). Results remained consistent when we considered CVD and non-CVD death as competing events (Supplementary Table 1). Significant trends were observed for all-cause, CVD and non-CVD death (p for trend < 0.01) (Table 2). The observed dose-response trends remained significant when we performed stratified analysis by follow-up duration (0–10 vs. 10 + years) (Supplementary Table 2). Further, when we excluded the first two years of follow-up, results remained virtually the same (results not shown). On the other hand, the associations were stronger among females compared to males for all-cause death as well as for CVD and non-CVD death (Supplementary Table 3).

**Table 2:** Hazard ratios (HRs) and 95% confidence interval (CIs) of death by the number of ideal cardiovascular health metrics

	Ideal health metrics, n	No. of cases	Person-years	Incidence rates (per1000)	HR (95% CI)	*P*-value for trend
All causes						
	<=2	279	14,207	10.98	**2.07 (1.46–2.92)** ^s,a^	< 0.0001 ^s,a^
	3	680	67,065	8.24	**1.59 (1.22–2.07)** ^s,a^	
	4	1,252	159,241	7.52	**1.65 (1.31–2.08)** ^s,a^	
	5	1,103	187,664	6.32	1.25 (0.99–1.58) ^s,a^	
	6–7	485	110,712	5.55	1.00 (Ref)	
CVD death						
	<=2	121	14,207	4.50	**3.24 (1.72–6.09)** ^s,a^	< 0.0001 ^s,a^
	3	246	67,065	3.10	**1.98 (1.19–3.30)** ^s,a^	
	4	408	159,241	2.46	**1.99 (1.26–3.17)** ^s,a^	
	5	350	187,664	1.97	1.43 (0.90–2.27) ^s,a^	
	6–7	136	110,712	1.62	1.00 (Ref)	
Non-CVD death						
	<=2	158	14,207	6.48	**2.08 (1.31–3.31)** ^e,a^	0.001 ^e,a^
	3	434	67,065	5.15	**1.61 (1.15–2.26)** ^e,a^	
	4	844	159,241	5.05	**1.51 (1.12–2.04)** ^e,a^	
	5	753	187,664	4.35	1.35 (0.99–1.83) ^e,a^	
	6–7	349	110,712	3.93	1.00 (Ref)	

CVD: cardiovascular diseases. Incidence rates are standardized for sex and age (5-year band)

Cox proportional hazards models are adjusted for age, sex and educational level. Cox models were stratified for the covariates that did not satisfy the PH assumption (s,a: stratified by sex and age. e,a: stratified by educational level and age)

Table 3 shows the HRs of death by each CVH metric separately (intermediate and poor vs. ideal). Intermediate and poor BMI had higher risk of all death outcomes compared with ideal BMI. Ideal metrics for physical activity, diabetes and blood pressure had significant lower risk of CVD death, and ideal metrics for non-smoking status had significant lower risk of non-CVD death. Consistent with the previous analysis, stronger associations were found among females also when each CVH metric was assessed separately. Interestingly, as expected, diabetes had a greater influence on CVD death among females (Females: HR = 4.25, *p* = 0.016 vs. males: HR = 2.22, *p* = 0.101) (Supplementary Table 4). When all 7 metrics were included in the Cox regression model, most of these factors remained significant, except BMI and diabetes in the analysis of CVD mortality and BMI in the non-CVD analysis (results not shown). When we included subjects with one missing of the 7 ideal CVH metrics (*n* = 35,812), individuals with poor or intermediate diet had a significantly higher risk of all-cause (HR = 1.22; 95% CI = 1.04–1.43) and non-CVD death (HR = 1.29; 95% CI = 1.04–1.60). When we evaluated the association between alcohol consumption and death, alcohol consumption did not increase the risk of death and no significant gains in life years were observed among individuals in the ideal alcohol category (Supplementary Table 5). When alcohol consumption was included in the ideal cardiovascular health metrics, results remained consistent, with increased risk of death as the number of ideal metrics decreases and with increasing life-year gain when the number of ideal metrics increases (Supplementary Table 5).

**Table 3:** Hazard ratios (HRs) and 95% confidence intervals (CIs) of death by each ideal cardiovascular health metric

Ideal health	All-cause death			CVD death			Non-CVD death		
	case, n	Person-years	HR (95% CI)	case, n	Person-years	HR (95% CI)	case, n	Person-years	HR (95% CI)
Smoking									
Intermediate or poor	1,415	192,621	**1.27(1.09–1.47)** ^s,a^	404	192,621	0.91(0.68–1.23) ^s,a^	1011	192,621	**1.32(1.09–1.60)** ^e,a^
Ideal	2,384	346,267	1.00(Ref)	857	346,267	1.00(Ref)	1527	346,267	1.00(Ref)
BMI									
Intermediate or poor	1,746	202,359	**1.27(1.10–1.46)** ^s,a^	608	202,359	**1.33(1.01–1.74)** ^s,a^	1138	202,359	**1.22(1.01–1.47)** ^e,a^
Ideal	2,053	336,529	1.00(Ref)	653	336,529	1.00(Ref)	1400	336,529	1.00(Ref)
Physical activity									
Intermediate or poor	2,767	349,537	1.11(0.88–1.41) ^s,a,e^	962	349,537	**1.50(1.10–2.05)** ^s,a^	1805	349,537	1.19(0.96–1.46) ^e,a^
Ideal	1,032	189,352	1.00(Ref)	299	189,352	1.00(Ref)	733	189,352	1.00(Ref)
Diet									
Intermediate or poor	3,316	475,057	1.19(0.97–1.45) ^s,a^	1099	475,057	1.17(0.79–1.74) ^s,a^	2217	475,057	1.30 (0.99–1.71) ^e,a^
Ideal	483	63,832	1.00(Ref)	162	63,832	1.00(Ref)	321	63,832	1.00(Ref)
Diabetes									
Poor (yes)	186	9,208	**1.73(1.17–2.54)** ^s,a^	80	9,208	**2.93(1.40–6.14)** ^s,a^	106	9,208	1.50 (0.91–2.50) ^e,a^
Ideal (no)	3,613	529,680	1.00(Ref)	1181	529,680	1.00(Ref)	2432	529,680	1.00(Ref)
Lipid disturbance									
Poor (yes)	256	13,601	0.95(0.70–1.29) ^s,a^	117	13,601	1.37(0.79–2.37) ^s,a^	139	13,601	0.80 (0.54–1.18) ^e,a^
Ideal (no)	3,543	525,288	1.00(Ref)	1144	525,288	1.00(Ref)	2399	525,288	1.00(Ref)
High blood pressure									
Poor (yes)	882	52,044	**1.37(1.15–1.64)** ^s,a^	398	52,044	**2.17 (1.56–3.01)** ^s,a^	484	52,044	1.06 (0.84–1.35) ^e,a^
Ideal (no)	2,917	486,844	1.00(Ref)	863	486,844	1.00(Ref)	2054	486,844	1.00(Ref)

CVD: cardiovascular diseases. Cox proportional hazards models are adjusted for age, sex and educational level. Cox models were stratified for the covariates that did not satisfy the PH assumption (s,a: Stratified by sex and age. s,a,e Stratified by sex, education level and age. e,a: Stratified by educational level and age.)

### Life-years gained

Laplace regression models showed that for participants with 6–7 CVH metrics, the median age at all-cause death, CVD and non-CVD death was respectively 4.19 (95% CI: 3.38;5.00), 5.77 (95% CI: 4.45;7.09) and 2.89 (95% CI: 1.78; 3.99) years higher compared to those with 0–2 CVH metrics for all causes, CVD and non-CVD death (Table 4). Results remained consistent when the analyses were stratified by sex (Supplementary Table 6). Moreover, significant differences in median age at death were observed for all CVH metrics in all-cause death analysis (absence vs. presence of diabetes: percentile difference [PD] = 3.25, 95% CI: 2.35–4.16; absence vs. presence of high blood pressure: PD = 1.98, 95% CI: 1.54–2.43), and for all CVH metrics except smoking and diet in CVD death analysis (absence vs. presence of diabetes: PD = 3.80, 95% CI: 2.53–5.07; absence vs. presence of high blood pressure: PD = 3.51, 95% CI = 2.87–4.15). We found significant median differences in non-CVD death analysis for smoking (PD = 1.46, 95% CI = 1.01–1.92), self-reported diabetes (PD = 2.18, 95% CI = 1.12–3.25) and hypertension (PD = 0.95, 95% CI = 0.42–1.48) (Supplementary Table 7). Generally, PD estimates were higher at 25th percentile of age at death and lower at 75th percentile, suggesting that the impact of CVH may be weaker among older individuals.

**Table 4:** Percentile Differences (PD) and 95% confidence interval (CIs) in Age at Death (years) according to the number of ideal cardiovascular health metrics

	Ideal health metrics, n	25th Percentile PD (CI)	50th Percentile PD (CI)	75th Percentile PD (CI)
All causes				
	<=2	0.00 (ref)	0.00 (ref)	0.00 (ref)
	3	**1.63(0.66;2.60)**	**1.96(1.17;2.75)**	**1.62(0.80;2.45)**
	4	**2.64(1.77;3.51)**	**2.53(1.81;3.26)**	**2.13(1.33;2.92)**
	5	**3.69(2.82;4.56)**	**3.37(2.64;4.10)**	**2.99(2.19;3.79)**
	6–7	**4.35(3.39;5.32)**	**4.19(3.38;5.00)**	**3.76(2.89; 4.64)**
CVD death				
	<=2	0.00 (ref)	0.00 (ref)	0.00 (ref)
	3	**2.75(1.36;4.15)**	**2.36(1.14;3.58)**	**1.38(0.28;2.48)**
	4	**3.79(2.48;5.11)**	**3.36(2.20;4.52)**	**2.61(1.55;3.67)**
	5	**4.96(3.64;6.29)**	**4.45(3.29;5.61)**	**3.79(2.70;4.89)**
	6–7	**5.89(4.38;7.40)**	**5.77(4.45;7.09)**	**4.93(3.67;6.19)**
Non-CVD death				
	<=2	0.00 (ref)	0.00 (ref)	0.00 (ref)
	3	1.05(-0.00; 2.10)	**1.28(0.22;2.34)**	0.94(-0.01; 1.89)
	4	**1.74(0.76;2.71)**	**1.59(0.59;2.59)**	**1.33(0.45; 2.22)**
	5	**2.48(1.49;3.46)**	**2.40(1.39;3.41)**	**2.08(1.18; 2.98)**
	6–7	**3.24(2.19;4.29)**	**2.89(1.78;3.99)**	**2.67(1.68; 3.65)**

Life-years gained. Estimates were obtained by conducting a Laplace regression on the 25th, 50th, and 75th percentiles of age at death, with number of ideal cardiovascular health metrics used as the main exposure and adjustment for age at baseline, sex and educational level

### ROC curve

**Fig. 1:**
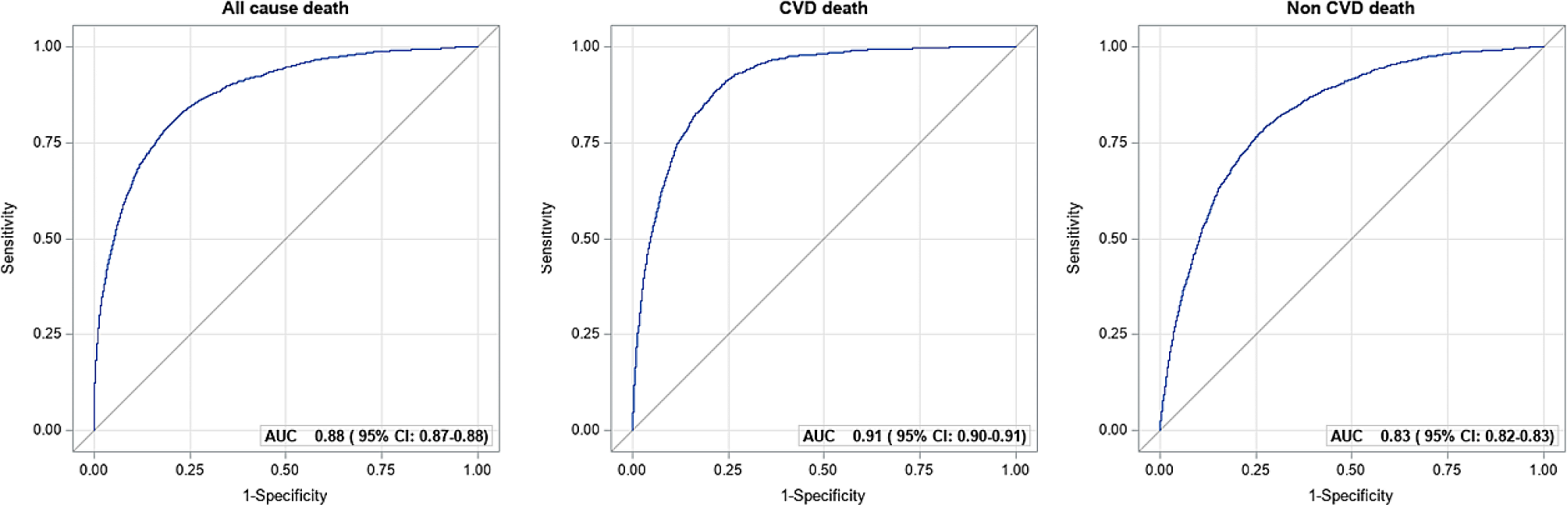
Receiver Operating Characteristic (ROC) Curve and area under the ROC curve (AUC). Prediction of all-cause death, CVD and non-CVD death based on the number of ideal CVH metrics, age at baseline, sex and education

When only ideal CVH metrics were considered, the AUCs were 0.60 (95% CI = 0.59–0.61) for predicting risk of all-cause death, 0.62 (95% CI = 0.60–0.63) for predicting CVD death and 0.58 (95% CI = 0.57–0.59) for predicting non-CVD death, respectively. After further including age, sex and education, the AUCs increased to 0.88 (95% CI = 0.87–0.88) for all-cause death, 0.91 (95% CI = 0.90–0.91) for CVD death, and 0.83 (95% CI = 0.82–0.83) for non-CVD death (Fig. 1). These AUCs, however, were only slightly higher compared to the prediction models based on only age, sex and education (0.87, 95% CI = 0.87–0.88; 0.90, 95% CI = 0.90–0.91; 0.82, 95% CI = 0.82–0.83, respectively).

### Interaction by age

We found significant interactions of the number of ideal CVH metrics with age at baseline continuously for the risk of all-cause death (*p* = 0.001), CVD death (*p* < 0.001) and non-CVD death (*p* = 0.013). Similarly, there were significant interactions with age as a categorical variable (< 65 and > = 65 years) for all-cause death (*p* = 0.002) and for CVD death (*p* < 0.001) (Supplementary Table 8).

Independent of age < 65 or ≥ 65, participants with 0–2 ideal metrics had a significantly higher risk of all cause, CVD and non-CVD death compared to those with 6–7 CVH metrics. Nonetheless, HRs were higher for participants below 65 years of age at baseline (HR = 2.24 vs. 1.94, HR = 3.58 vs. 3.10 and HR = 2.39 vs. 1.43 respectively for all causes, CVD and non-CVD death) (Supplementary Table 9). For participants with 3, 4 or 5 CVH metrics, significantly higher risks of CVD death were observed among participants > = 65, but not for those < 65 years, while the pattern was the opposite for non-CVD death. When the additive interactions were assessed, we found a significant relative excess risk of death due to interaction for < = 5 vs. 6–7 ideal health metrics (*p* = 0.021) and significant relative excess risk of CVD death due to interaction for < = 3 vs. 4+, <=4 vs. 5 + and < = 5 vs. 6–7 ideal health metrics (respectively *p* = 0.002, *p* = 0.006, *p* < 0.001) (results not shown).

In the Laplace regression analyses, we found significant interactions between the number of ideal CVH metrics and age at baseline for all-cause mortality (25th percentile: *p* = 0.0018; 50th percentile: *p* = 0.0108; 75th percentile: *p* = 0.0491) and for non-CVD death (25th percentile *p* = 0.0442; *p* = 0.0407; *p* = 0.0198). We showed results separately for individuals with baseline ages < 65 and ≥ 65 years (Supplementary Table 10). The patterns observed in age-stratified Cox analyses were confirmed.

## Discussion

Our analysis of the Swedish National March Cohort reveals a significant inverse association of number of ideal CVH metrics with risk of death in this population with a low prevalence of ideal cardiovascular health metrics. Moreover, compared with participants characterized by 0–2 CVH metrics, among those with 6–7 ideal metrics the median age at death was extended by 4.2 years for all-causes, 5.8 years for CVD and 2.9 years for non-CVD.

We found a low prevalence of ideal cardiovascular health in our cohort. Out of 29,557 participants aged 18–94, only 2% met all 7 ideal health metrics. In one cross-sectional Danish study – including only smoking, BMI, blood pressure, total cholesterol and diabetes as the definition of ideal health - the proportion with ideal cardiovascular health increased from 1.6% in 1978 to 9% in 2006 [29]. Low prevalence of ideal cardiovascular health was also found in other national and regional populations [4], such as in the US [30], UK [9], Spain [31] and China [32]. The Framingham offspring study revealed a decrease in the percentage of people with ideal CVH over the past 20 years [33]. As the baseline of our study was conducted in 1997, the prevalence of ideal CVH in Sweden might not represent the current situation. Although a general population cohort of people was assembled for the Swedish National March Cohort, the total number of individuals who in reality were given a questionnaire could not be assessed, thus the prevalence of ideal CVH in this study does not represent the national situation.

A significant inverse association of number of ideal CVH metrics with risk of death was observed for all-cause, CVD and non-CVD death, after adjusting for the potential confounding factors. Previous studies have shown an inverse association of ideal CVH with incidence of death or CVD-related death. A meta-analysis including 6 studies indicated a linear decrease in all-cause mortality, with a pooled HR of 0.89 for each unit increment of ideal CVH metrics [13]. The ARIC study also provided evidence that the adherence to ideal CVH was associated with lower lifetime risk of heart disease [34]. In our study, participants with lower CVH had higher risk of death compared to subjects in the highest CVH category, which is consistent with previous studies. We additionally found this association after 10- and 10 + years of follow-up, which supports use of CVH for lifelong health promotion. In practice, behaviour changing of smoking, diet, or physical activity has been validated to lower the risk of chronic diseases, for example, a wearable use of smart product to increase the physical activity participation could obtain a healthy cardiorespiratory fitness [35].

We found that ideal CVH was also associated with non-cardiovascular disease death. A cohort study conducted in the US showed a lower risk of cancer in association with ideal CVH, which in part supports our findings for non-CVD death [36]. Moreover, several studies have identified an inverse association of ideal CVH with risk of hyperuricemia, proteinuria, insulin resistance and non-alcohol fatty liver [14, 15, 37, 38]. Hence, a reduction of adverse levels of risk factors before the first occurrence of clinical events might promote health for the whole population [39, 40]. In particular, significantly higher risks of non-CVD death were observed among younger adults (participants < 65 compared to > = 65 years), suggesting that the promotion of ideal CVH in young adults might be more helpful for the prevention of non-CVD.

Our study has several limitations. First, the definition of ideal health factors is based on self-report rather than clinical or laboratory data, which may entail misclassification of exposures, for example some individuals may have been undiagnosed with hypertension, diabetes, and hypercholesterolemia. Thus the prevalence of diabetes, lipid disturbance and hypertension might likely be underestimated in absence of laboratory markers and medication data [41, 42]. Furthermore, the definition of smoking is never, former and current smoking, thus the definition of ideal cardiovascular health is not identical to the AHA’s definition. Second, prevalence of CVH factors refers to 20 years ago, and the definition of what constituted hypertension and hypercholesterolemia was different 20 years ago. Therefore, we do not know if the prevalence of ideal health has improved in recent years. Moreover, the percentage of participants adhering to physical activity recommendations was generally high and the prevalence of smoking and alcohol drinking were relative low in this study, which limits the external validity of the results. Third, we only had baseline data, thus possible changes in lifestyle during follow-up could not be assessed. Furthermore, excluding participants with missing risk factors might result bias, although sensitivity analysis shows similar results when including subjects with one missing of the 7 CVH factors. All these limitations would most likely lead to misclassification bias and might entail underestimation of any true association between CVH categories and risk of death.

This study is one of largest cohort studies conducted in Nordic countries which aims to explore the association of ideal CVH with the risk of all-cause, CVD or non-CVD death, with long-term follow-up period. Strengths of our study include the large sample size, prospective design, a detailed questionnaire on lifestyle, and complete long-term follow-up due to the use of personal registration number and completeness of national demographic and health registers. By adopting Laplace regression method, we further identified the extended median age at death for participants with more ideal metrics, suggesting even a moderate or small improvement of lifestyle could contribute to reducing deaths for the whole population.

## Conclusions

Our findings indicated a strong inverse association of ideal CVH with risk of all-cause, CVD or non-CVD death with a median age at death that was significantly higher among those with 6–7 ideal metrics compared to those with 0–2 CVH metrics. Our study supports the application of ideal CVH for individual risk assessment and health promotion for the general population.

### Electronic supplementary material

Below is the link to the electronic supplementary material.

Supplementary Material 1

Supplementary Material 2

## Data Availability

The datasets used and/or analyzed during the current study are available from the corresponding author on reasonable request.

## Abbreviations

AHA: American Heart Association
AUC: Area under the ROC curve
CVD: Cardiovascular diseases
CVH: Cardiovascular health
FFQ: Food frequency questionnaire
HR: Hazards ratio
PD: Percentile difference
PH: Proportional-hazards
ROC: Receiver Operating Characteristic
SD: Standard deviation
