# Supplementary material for: Ideal cardiovascular health and risk of death in a large Swedish cohort
Source: BMC Public Health. 2024 Feb 2;24:358. doi: 10.1186/s12889-024-17885-4 (PMC10837860; doi:10.1186/s12889-024-17885-4)
Supplement: Supplementary file 1 — Supplementary Material 1 [file 12889_2024_17885_MOESM1_ESM.pptx]

## Slide 1
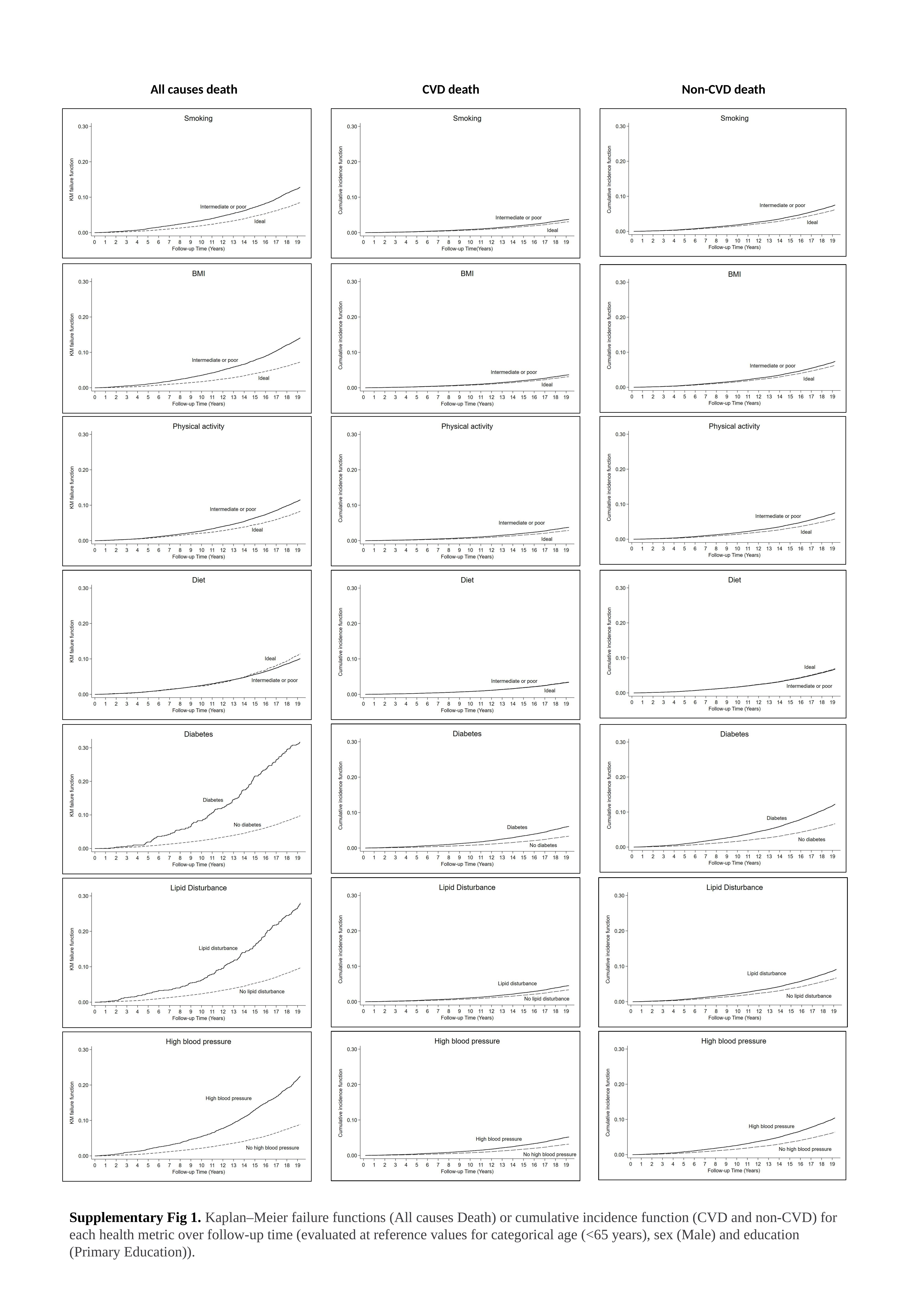

All causes death CVD death Non-CVD death
Supplementary Fig 1. Kaplan–Meier failure functions (All causes Death) or cumulative incidence function (CVD and non-CVD) for each health metric over follow-up time (evaluated at reference values for categorical age (<65 years), sex (Male) and education (Primary Education)).

## Slide 2
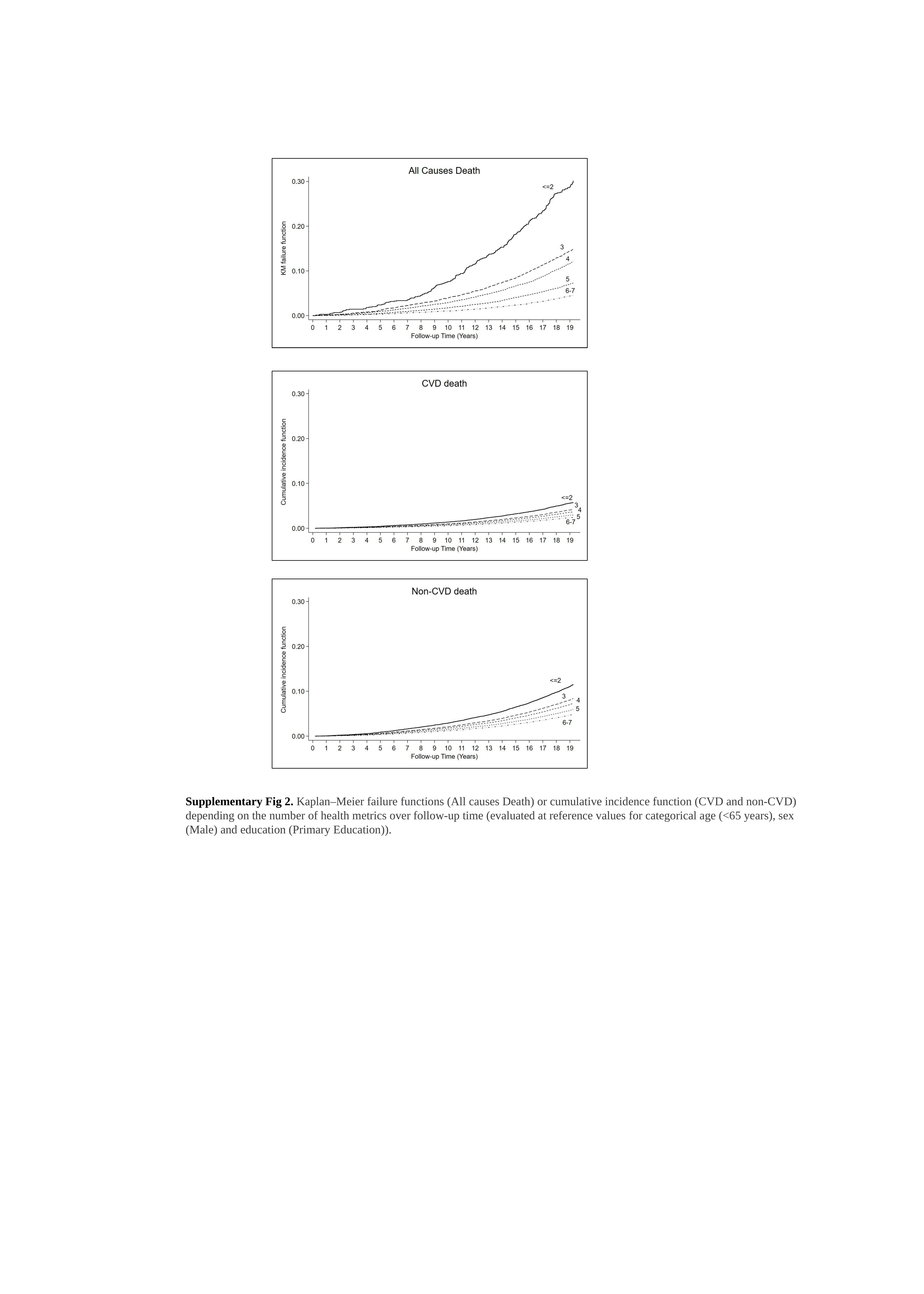

Supplementary Fig 2. Kaplan–Meier failure functions (All causes Death) or cumulative incidence function (CVD and non-CVD) depending on the number of health metrics over follow-up time (evaluated at reference values for categorical age (<65 years), sex (Male) and education (Primary Education)).
