# Supplementary material for: Ideal cardiovascular health and risk of death in a large Swedish cohort
Source: BMC Public Health. 2024 Feb 2;24:358. doi: 10.1186/s12889-024-17885-4 (PMC10837860; doi:10.1186/s12889-024-17885-4)
Supplement: Supplementary file 2 — Supplementary Material 2 [file 12889_2024_17885_MOESM2_ESM.docx]

**Supplementary Material**

**Supplementary Table 1.** SHRs and 95% CIs of CVD and Non-CVD death considering the two types of death as competing risks. Fine-Gray models were performed and adjusted for age, sex and education.

| **Ideal health** | **CVD death** | **Non-CVD death** |
| --- | --- | --- |
|  | **SHR (95% CI)** | **SHR (95% CI)** |
| **Smoking** |  |  |
| Intermediate or poor | 0.99(0.87-1.13) | **1.32(1.22-1.44)** |
| Ideal | 1.00(Ref) | 1.00(Ref) |
| **BMI** |  |  |
| Intermediate or poor | **1.31(1.17-1.47)** | 1.07(0.99-1.16) |
| Ideal | 1.00(Ref) | 1.00(Ref) |
| **Physical activity** |  |  |
| Intermediate or poor | **1.32(1.15-1.51)** | 1.02(0.94-1.12) |
| Ideal | 1.00(Ref) | 1.00(Ref) |
| **Diet** |  |  |
| Intermediate or poor | 0.98(0.83-1.16) | 1.10 (0.98-1.24) |
| Ideal | 1.00(Ref) | 1.00(Ref) |
| **Diabetes** |  |  |
| Poor (yes) | **2.07(1.60-2.68)** | **1.33 (1.08-1.64)** |
| Ideal (no) | 1.00(Ref) | 1.00(Ref) |
| **Lipid disturbance** |  |  |
| Poor (yes) | **1.75(1.44-2.13)** | 1.03 (0.86-1.23) |
| Ideal (no) | **1.00(Ref)** | 1.00(Ref) |
| **High blood pressure** |  |  |
| Poor (yes) | **2.02 (1.78-2.29)** | **1.11 (1.00-1.23)** |
| Ideal (no) | 1.00(Ref) | 1.00(Ref) |
| **Ideal health metrics, N** |  |  |
| <=2 | **2.80(2.16-3.62)** | **1.54(1.27-1.87)** |
| 3 | **1.85(1.49-2.30)** | **1.30(1.13-1.50)** |
| 4 | **1.54(1.26-1.88)** | **1.25(1.10-1.42)** |
| 5 | 1.19(0.97-1.46) | 1.08(0.95-1.22) |
| 6-7 | 1.00(Ref) | 1.00(Ref) |

**Supplementary Table 2***.* Hazard ratios (HRs) and 95% confidence interval (CIs) of death by the number of ideal cardiovascular health metrics, stratified by follow-up duration

|  | 0-10 years of follow-up | | | | 10+ years of follow-up | | | |
| --- | --- | --- | --- | --- | --- | --- | --- | --- |
|  | Ideal health metrics, n | No. of cases | HR (95% CI) | P-value for trend | Ideal health metrics, n | No. of cases | HR (95% CI) | P-value for trend |
| All causes |  |  |  | 0.008 ^s,a^ |  |  |  | <0.001^a^ |
|  | <=2 | 78 | 1.67(0.89-3.14)^s,a^ |  | <=2 | 201 | 2.68(1.94-3.70) ^a^ |  |
|  | 3 | 203 | 1.45(0.90-2.34) ^s,a^ |  | 3 | 477 | 1.68(1.32-2.15) ^a^ |  |
|  | 4 | 334 | 1.51(0.99-2.31) ^s,a^ |  | 4 | 918 | 1.66(1.34-2.06) ^a^ |  |
|  | 5 | 308 | 0.99(0.65-1.52) ^s,a^ |  | 5 | 795 | 1.37(1.10-1.71) ^a^ |  |
|  | 6-7 | 122 | 1.00 (Ref) |  | 6-7 | 363 | 1.00 (Ref) |  |
| CVD death |  |  |  | 0.009 ^s,a^ |  |  |  | <0.001 |
|  | <=2 | 45 | 2.45(0.83-7.25) ^s,a^ |  | <=2 | 76 | 2.87(2.12-3.89) |  |
|  | 3 | 71 | 1.67(0.62-4.48) ^s,a^ |  | 3 | 175 | 2.08(1.62-2.67) |  |
|  | 4 | 104 | 1.30(0.54-3.14) ^s,a^ |  | 4 | 304 | 1.70(1.36-2.14) |  |
|  | 5 | 80 | 0.68(0.28-1.64) ^s,a^ |  | 5 | 270 | 1.38(1.10-1.74) |  |
|  | 6-7 | 37 | 1.00 (Ref) |  | 6-7 | 99 | 1.00 (Ref) |  |
| Non-CVD death |  |  |  | 0.003 |  |  |  | <0.001 ^a^ |
|  | <=2 | 33 | 1.32(0.88-1.98) |  | <=2 | 125 | 2.39(1.63-3.50) ^a^ |  |
|  | 3 | 132 | 1.63(1.23-2.15) |  | 3 | 302 | 1.60(1.20-2.12) ^a^ |  |
|  | 4 | 230 | 1.41(1.10-1.82) |  | 4 | 614 | 1.60(1.25-2.06) ^a^ |  |
|  | 5 | 228 | 1.31(1.02-1.69) |  | 5 | 525 | 1.30(1.00-1.67) ^a^ |  |
|  | 6-7 | 85 | 1.00 (Ref) |  | 6-7 | 264 | 1.00 (Ref) |  |

CVD: cardiovascular diseases.

Cox proportional hazards models are adjusted for age, sex and educational level. Cox models were stratified for the covariates that did not satisfy the PH assumption (s,a: Stratified by sex and age. a: Stratified by age).

**Supplementary Table 3***.* Hazard ratios (HRs) and 95% confidence interval (CIs) of death by the number of ideal cardiovascular health metrics, among males and females.

|  | *Males* | | | *Females* | | |
| --- | --- | --- | --- | --- | --- | --- |
|  | Ideal health metrics, n | HR (95% CI) | P-value for trend | Ideal health metrics, n | HR (95% CI) | P-value for trend |
| All causes |  |  | 0.111 ^a^ |  |  | **<0.001** ^a^ |
|  | <=2 | **1.66(1.02-2.71)** ^a^ |  | <=2 | **2.67(1.62-4.39)** ^a^ |  |
|  | 3 | 1.21(0.81-1.79) ^a^ |  | 3 | **2.02(1.42-2.89)** ^a^ |  |
|  | 4 | 1.35(0.94-1.94) ^a^ |  | 4 | **1.93(1.42-2.61)** ^a^ |  |
|  | 5 | 1.27(0.86-1.86) ^a^ |  | 5 | 1.31(0.97-1.77) ^a^ |  |
|  | 6-7 | 1.00 (Ref) |  | 6-7 | 1.00 (Ref) |  |
| CVD death |  |  | 0.075 ^a^ |  |  | **<0.001** ^a^ |
|  | <=2 | 2.18(0.95-5.02) ^a^ |  | <=2 | **5.60(2.11-14.81)** ^a^ |  |
|  | 3 | 1.29(0.65-2.56) ^a^ |  | 3 | **3.67(1.65-8.15)** ^a^ |  |
|  | 4 | 1.86(0.97-3.58) ^a^ |  | 4 | **2.38(1.21-4.69)** ^a^ |  |
|  | 5 | 1.14(0.58-2.27) ^a^ |  | 5 | 1.90(0.98-3.69) ^a^ |  |
|  | 6-7 | 1.00 (Ref) |  | 6-7 | 1.00 (Ref) |  |
| Non-CVD death |  |  | 0.517 ^a^ |  |  | **<0.001** ^a^ |
|  | <=2 | 1.41(0.77-2.59) ^a^ |  | <=2 | **2.01(1.11-3.64)** ^a^ |  |
|  | 3 | 1.19 (0.72-1.94) ^a^ |  | 3 | **1.71(1.15-2.54)** ^a^ |  |
|  | 4 | 1.17 (0.75-1.81) ^a^ |  | 4 | **1.83(1.30-2.58)** ^a^ |  |
|  | 5 | 1.32(0.83-2.11) ^a^ |  | 5 | 1.18(0.84-1.66) ^a^ |  |
|  | 6-7 | 1.00 (Ref) |  | 6-7 | 1.00 (Ref) |  |

CVD: cardiovascular diseases.

Cox proportional hazards models were adjusted for age and educational level. Cox models were stratified for the covariates that did not satisfy the PH assumption (a: Stratified by age).

**Supplementary Table 4**. Hazard ratios (HRs) and 95% confidence intervals (CIs) of death by each ideal cardiovascular health metric, among males and females.

|  | **All causes** | | **CVD death** | | **Non CVD death** | |
| --- | --- | --- | --- | --- | --- | --- |
|  | ***Males*** | ***Females*** | ***Males*** | ***Females*** | ***Males*** | ***Females*** |
| **Smoking** | **HR(95% CI)** | **HR(95% CI)** | **HR(95% CI)** | **HR(95% CI)** | **HR(95% CI)** | **HR(95% CI)** |
| **Intermediate or poor** | 1.05(0.84-1.31)^a^ | **1.48(1.21-1.80)**^a^ | 0.91(0.61-1.35)^a^ | 0.92(0.59-1.44)^a^ | 1.12(0.85-1.47)^a^ | **1.68(1.34-2.10)**^a^ |
| **Ideal** | 1.00(Ref) | 1.00(Ref) | 1.00(Ref) | 1.00(Ref) | 1.00(Ref) | 1.00(Ref) |
| **BMI** |  |  |  |  |  |  |
| **Intermediate or poor** | 1.17(0.93-1.46)^a^ | **1.35(1.13-1.62)**^a^ | 1.25(0.84-1.87)^a^ | 1.40(0.96-2.04)^a^ | 1.12(0.85-1.48)^a^ | **1.34(1.08-1.65)**^a^ |
| **Ideal** | 1.00(Ref) | 1.00(Ref) | 1.00(Ref) | 1.00(Ref) | 1.00(Ref) | 1.00(Ref) |
| **Physical activity** |  |  |  |  |  |  |
| **Intermediate or poor** | 1.05(0.82-1.34)^a^ | 1.11(0.81-1.52)^a,e^ | 1.18(0.77-1.80)^a^ | **1.99(1.25-3.17)**^a^ | 0.98(0.73-1.33)^a^ | 0.94(0.74-1.20)^a^ |
| **Ideal** | 1.00(Ref) | 1.00(Ref) | 1.00(Ref) | 1.00(Ref) | 1.00(Ref) | 1.00(Ref) |
| **Diet** |  |  |  |  |  |  |
| **Intermediate or poor** | 1.08(0.77-1.50)^a^ | 1.26(0.98-1.62)^a^ | 0.90(0.49-1.67)^a^ | 1.44(0.85-2.41)^a^ | 1.15 (0.77-1.72)^a^ | 1.21(0.91-1.62)^a^ |
| **Ideal** | 1.00(Ref) | 1.00(Ref) | 1.00(Ref) | 1.00(Ref) | 1.00(Ref) | 1.00(Ref) |
| **Diabetes** |  |  |  |  |  |  |
| **Poor (yes)** | 1.61(0.94-2.76)^a^ | **1.87(1.08-3.25)**^a^ | 2.22(0.85-5.78)^a^ | **4.25(1.31-13.80**)^a^ | 1.38 (0.71-2.67)^a^ | 1.41(0.75-2.68)^a^ |
| **Ideal (no)** | 1.00(Ref) | 1.00(Ref) | 1.00(Ref) | 1.00(Ref) | 1.00(Ref) | 1.00(Ref) |
| **Lipid disturbance** |  |  |  |  |  |  |
| **Poor (yes)** | 0.86(0.54-1.36)^a^ | 1.03(0.68-1.55)^a^ | 1.34(0.60-3.02)^a^ | 1.36(0.65-2.87)^a^ | 0.69(0.39-1.22)^a^ | 0.91(0.55-1.49)^a^ |
| **Ideal (no)** | 1.00(Ref) | 1.00(Ref) | 1.00(Ref) | 1.00(Ref) | 1.00(Ref) | 1.00(Ref) |
| **High blood pressure** |  |  |  |  |  |  |
| **Poor (yes)** | 1.25(0.94-1.65)^a^ | **1.46(1.17-1.83)**^a^ | **2.01(1.22-3.31)**^a^ | **2.29(1.48; 3.55)**^a^ | 0.98(0.69-1.39)^a^ | 1.23(0.94-1.60)^a^ |
| **Ideal (no)** | 1.00(Ref) | 1.00(Ref) | 1.00(Ref) | 1.00(Ref) | 1.00(Ref) | 1.00(Ref) |

CVD: cardiovascular diseases. Cox proportional hazards models are adjusted for age and educational level. Cox models were stratified for the covariates that did not satisfy the PH assumption (a: Stratified by age; a,e Stratified by education level and age)

**Supplementary Table 5.** Hazard ratios (HRs) and 95% confidence interval (CIs) of death by alcohol consumption and by the number of ideal cardiovascular health metrics including alcohol consumption. Percentile Differences (PD) and 95% confidence interval (CIs) in Age at Death (years) according to the alcohol consumption and to the number of ideal cardiovascular health metrics including alcohol consumption.

|  |  | **HR (95% CI)** | **P-value for trend** | **25^th^ Percentile**  **PD (CI)** | **50^th^ Percentile**  **PD (CI)** | **75^th^ Percentile**  **PD (CI)** |
| --- | --- | --- | --- | --- | --- | --- |
| **All causes** | Ideal health metrics, n |  |  |  |  |  |
|  | <=2 | **3.59 (2.19-5.87)**^a^ | **<0.001**^a^ | 0.00 (ref) | 0.00 (ref) | 0.00 (ref) |
|  | 3 | **2.16 (1.66-2.82)**^a^ |  | 0.79 (-1.11; 2.70) | 1.11 (-0.23; 2.44) | 1.25(-0.16; 2.67) |
|  | 4 | **1.51 (1.22 -1.86)**^a^ |  | **2.57 (0.73; 4.41)** | **2.62 (1.41; 3.82)** | **2.01(0.73; 3.29)** |
|  | 5 | **1.47 (1.21-1.78)**^a^ |  | **3.34 (1.54; 5.15)** | **3.24 (2.06; 4.41)** | **2.67(1.40; 3.94)** |
|  | 6 | 1.21 (0.99; 1.48)^a^ |  | **4.18 (2.37; 5.99)** | **3.96 (2.78; 5.13)** | **3.41(2.13; 4.69)** |
|  | 7-8 | 1.00 (Ref) |  | **5.05 (3.19; 6.91)** | **4.80 (3.58; 6.03)** | **4.08(2.75; 5.41)** |
|  | Alcohol Consumption |  |  |  |  |  |
|  | Not Ideal | 0.86(0.74; 1.00)^a^ | --- | 0.00 (ref) | 0.00 (ref) | 0.00 (ref) |
|  | Ideal | 1.00(ref) |  | -0.46(-0.97; 0.06) | -0.28(-0.74; 0.19) | 0.03(-0.43; 0.50) |
| **CVD death** | Ideal health metrics, n |  |  |  |  |  |
|  | <=2 | **4.56 (1.55-13.44)** ^a,s^ | **<0.001 ^a,s^** | 0.00 (ref) | 0.00 (ref) | 0.00 (ref) |
|  | 3 | **3.02 (1.58-5.79)** ^a,s^ |  | 1.68 (-0.59; 3.96) | 1.78 (-0.20; 3.75) | **1.98 (0.27; 3.69)** |
|  | 4 | **1.77 (1.04-3.02)** ^a,s^ |  | **3.36 (1.30; 5.43)** | **2.78 (1.03; 4.53)** | **2.93 (1.28; 4.57)** |
|  | 5 | **1.89 (1.15-3.10)** ^a,s^ |  | **4.64 (2.60; 6.68)** | **3.98 (2.26; 5.70)** | **4.09 (2.46; 5.71)** |
|  | 6 | 1.44 (0.87; 2.40) ^a,s^ |  | **5.57 (3.51; 7.63)** | **5.02 (3.27; 6.76)** | **5.30 (3.65; 6.95)** |
|  | 7-8 | 1.00 (Ref) |  | **6.64 (4.44; 8.84)** | **6.05 (4.19; 7.90)** | **6.26 (4.45; 8.08)** |
|  | Alcohol Consumption |  |  |  |  |  |
|  | Not Ideal | 1.05 (0.74; 1.49) ^a,s^ | --- | 0.00 (ref) | 0.00 (ref) | 0.00 (ref) |
|  | Ideal | 1.00(ref) |  | -0.50 (-1.27; 0.27) | -0.17 (-0.88; 0.53) | -0.11 (-0.80; 0.58) |
| **Non-CVD death** | Ideal health metrics, n |  |  |  |  |  |
|  | <=2 | **2.72 (1.46-5.08)** ^a^ | **<0.001 ^a^** | 0.00 (ref) | 0.00 (ref) | 0.00 (ref) |
|  | 3 | **1.93 (1.41-2.63)** ^a^ |  | 0.14 (-1.98; 2.27) | -0.09 (-1.94; 1.76) | 0.42 (-1.20; 2.04) |
|  | 4 | **1.46 (1.14-1.86)** ^a^ |  | 1.46 (-0.60; 3.52) | 1.05 (-0.69; 2.78) | 1.41 (-0.12; 2.93) |
|  | 5 | 1.42 (1.14-1.78) ^a^ |  | 1.84 (-0.18; 3.87) | 1.41 (-0.30; 3.13) | **1.72 (0.22; 3.21)** |
|  | 6 | 1.18 (0.93; 1.49) ^a^ |  | **2.52 (0.48; 4.56)** | **2.06 (0.33; 3.79)** | **2.35 (0.84; 3.87)** |
|  | 7-8 | 1.00 (Ref) |  | **3.40 (1.32; 4.59)** | **2.57 (0.78; 4.36)** | **2.95 (1.36; 4.54)** |
|  | Alcohol Consumption |  |  |  |  |  |
|  | Not Ideal | 0.83(0.70; 0.99) ^a^ | --- | 0.00 (ref) | 0.00 (ref) | 0.00 (ref) |
|  | Ideal | 1.00(ref) |  | -0.16 (-0.77; 0.45) | -0.05 (-0.63; 0.53) | 0.03 (-0.51; 0.56) |

CVD: cardiovascular diseases.

Cox proportional hazards models are adjusted for age, sex and educational level. Cox models were stratified for the covariates that did not satisfy the PH assumption (a: stratified by age, a,s: stratified by sex and age); PD estimates were obtained by conducting a Laplace regression on the 25th, 50th, and 75th percentiles of age at death adjusting for age at baseline, sex and educational level.

**Supplementary Table 6**. Percentile Differences (PD) and 95% confidence interval (CIs) in age at death (years) according to the number of ideal cardiovascular health metrics among males and females.

|  |  | *Males* | | | *Females* | | |
| --- | --- | --- | --- | --- | --- | --- | --- |
|  | Ideal health metrics, n | 25^th^ Percentile  PD (CI) | 50^th^ Percentile  PD (CI) | 75^th^ Percentile  PD (CI) | 25^th^ Percentile  PD (CI) | 50^th^ Percentile  PD (CI) | 75^th^ Percentile  PD (CI) |
| All causes |  |  |  |  |  |  |  |
|  | <=2 | 0.00 (ref) | 0.00 (ref) | 0.00 (ref) | 0.00 (ref) | 0.00 (ref) | 0.00 (ref) |
|  | 3 | **1.51(0.14;2.87)** | **1.98(0.92;3.04)** | **1.37(0.10;2.64)** | **1.59(0.22;2.96)** | **1.92(0.77;3.07)** | **1.69(0.65;2.73)** |
|  | 4 | **3.01(1.76;4.26)** | **3.07(2.08;4.05)** | **2.25(1.00;3.50)** | **2.06(0.82;3.29)** | **1.97(0.92;3.02)** | **1.96(0.98;2.93)** |
|  | 5 | **3.97(2.74;5.19)** | **3.64(2.66;4.62)** | **2.92(1.66;4.19)** | **3.17(1.93;4.41)** | **3.03(1.97;4.09)** | **2.95(1.97;3.93)** |
|  | 6-7 | **4.30(2.91;5.69)** | **4.75(3.61;5.89)** | **3.97(2.63; 5.30)** | **4.06(2.73;5.40)** | **3.56(2.43;4.70)** | **3.49(2.33; 4.65)** |
| CVD death |  |  |  |  |  |  |  |
|  | <=2 | 0.00 (ref) | 0.00 (ref) | 0.00 (ref) | 0.00 (ref) | 0.00 (ref) | 0.00 (ref) |
|  | 3 | **3.12(1.13; 5.12)** | **2.36(0.49;4.24)** | 1.24(-0.17; 2.65) | **1.98(0.33; 3.63)** | **2.50(1.03; 3.97)** | **2.18(0.68; 3.68)** |
|  | 4 | **4.51(2.63;6.40)** | **3.50(1.68;5.31)** | **2.55(1.19; 3.90)** | **2.77(1.27; 4.27)** | **3.19(1.81; 4.58)** | **3.15(1.72; 4.58)** |
|  | 5 | **5.64(3.75;7.53)** | **4.74(2.88;6.60)** | **3.72(2.36; 5.09)** | **3.91(2.38; 5.43)** | **4.23(2.87; 5.59)** | **4.03(2.59; 5.47)** |
|  | 6-7 | **6.44(4.31;8.58)** | **5.95(3.92;7.98)** | **4.64(3.03; 6.25)** | **4.74(2.92; 6.56)** | **5.67(3.97; 7.38)** | **5.70(3.90; 7.50)** |
| Non-CVD death |  |  |  |  |  |  |  |
|  | <=2 | 0.00 (ref) | 0.00 (ref) | 0.00 (ref) | 0.00 (ref) | 0.00 (ref) | 0.00 (ref) |
|  | 3 | 0.85(-0.40; 2.10) | 1.20(-0.32; 2.72) | 0.60(-0.62; 1.83) | 1.43(-0.49; 3.35) | 1.11(-0.44; 2.65) | 1.43(-0.10; 2.95) |
|  | 4 | **1.82(0.65; 2.99)** | **1.95(0.46; 3.43)** | **1.41(0.26; 2.56)** | 1.53(-0.21; 3.27) | 0.98(-0.43; 2.40) | 1.34(-0.07; 2.76) |
|  | 5 | **2.48(1.32; 3.64)** | **2.43(0.92; 3.94)** | **1.87(0.71; 3.03)** | **2.44(0.68; 4.20)** | **2.08(0.65; 3.52)** | **2.40(0.97; 3.83)** |
|  | 6-7 | **3.46(2.09; 4.83)** | **3.33(1.72; 4.94)** | **2.75(1.45; 4.04)** | **2.98(1.16; 4.81)** | **2.29(0.70; 3.87)** | **2.69(1.13; 4.24)** |

Estimates were obtained by conducting a Laplace regression on the 25th, 50th, and 75th percentiles of age at death, with number of ideal cardiovascular health metrics used as the main exposure and adjustment for age at baseline and educational level.

**Supplementary Table 7***.* Percentile Differences (PD) and 95% confidence interval (CIs) in Age at Death (years) according to each single ideal cardiovascular health metric.

| Ideal health | All-cause death | | | CVD death | | | Non-CVD death | | |
| --- | --- | --- | --- | --- | --- | --- | --- | --- | --- |
|  | 25^th^ Percentile  PD (CI) | 50^th^ Percentile  PD (CI) | 75^th^ Percentile  PD (CI) | 25^th^ Percentile  PD (CI) | 50^th^ Percentile  PD (CI) | 75^th^ Percentile  PD (CI) | 25^th^ Percentile  PD (CI) | 50^th^ Percentile  PD (CI) | 75^th^ Percentile  PD (CI) |
| Smoking |  |  |  |  |  |  |  |  |  |
| Intermediate or poor | 0.00 (ref) | 0.00 (ref) | 0.00 (ref) | 0.00 (ref) | 0.00 (ref) | 0.00 (ref) | 0.00 (ref) | 0.00 (ref) | 0.00 (ref) |
| Ideal | **1.17(0.75; 1.59)** | **1.12(0.74; 1.50)** | **0.99(0.62; 1.36)** | 0.33(-0.30; 0.95) | 0.39(-0.24; 1.03) | 0.37(-0.25; 0.99) | **1.68(1.18;2.18)** | **1.46(1.01;1.92)** | **.1.40(0.98;1.82)** |
| BMI |  |  |  |  |  |  |  |  |  |
| Intermediate or poor | 0.00 (ref) | 0.00 (ref) | 0.00 (ref) | 0.00 (ref) | 0.00 (ref) | 0.00 (ref) | 0.00 (ref) | 0.00 (ref) | 0.00 (ref) |
| Ideal | **0.73(0.31; 1.14)** | **0.60(0.25; 0.96)** | **0.60(0.26; 0.95)** | **1.26(0.68; 1.84)** | **1.17(0.62; 1.72)** | **1.18(0.62; 1.74)** | 0.45(-0.15;0.91) | 0.38(-0.05;0.80) | 0.32(-0.10;0.73) |
| Physical activity |  |  |  |  |  |  |  |  |  |
| Intermediate or poor | 0.00 (ref) | 0.00 (ref) | 0.00 (ref) | 0.00 (ref) | 0.00 (ref) | 0.00 (ref) | 0.00 (ref) | 0.00 (ref) | 0.00 (ref) |
| Ideal | **0.79(0.33; 1.25)** | **0.75(0.36; 1.14)** | **0.76(0.35; 1.17)** | **1.48(0.79;2.18)** | **1.59(0.90; 2.28)** | **1.61(0.95; 2.26)** | 0.52(0.01;1.02) | 0.35(-0.13;0.83) | 0.39(-0.07;0.85) |
| Diet |  |  |  |  |  |  |  |  |  |
| Intermediate or poor | 0.00 (ref) | 0.00 (ref) | 0.00 (ref) | 0.00 (ref) | 0.00 (ref) | 0.00 (ref) | 0.00 (ref) | 0.00 (ref) | 0.00 (ref) |
| Ideal | **0.56(0.01; 1.10)** | **0.63(0.08; 1.17)** | 0.41(-0.07; 0.90) | 0.11(-0.84;1.06) | 0.33(-0.48;1.15) | 0.31(-0.53;1.15) | **0.88(0.21;1.55)** | 0.57(-0.05;1.18) | 0.55(-0.08;1.17) |
| Diabetes |  |  |  |  |  |  |  |  |  |
| Poor(yes) | 0.00 (ref) | 0.00 (ref) | 0.00 (ref) | 0.00 (ref) | 0.00 (ref) | 0.00 (ref) | 0.00 (ref) | 0.00 (ref) | 0.00 (ref) |
| Ideal (no) | **3.46(2.30; 4.61)** | **3.25(2.35; 4.16)** | **2.62(1.83; 3.41)** | **4.62(3.21;6.03)** | **3.80(2.53;5.07)** | **3.47(2.24;4.69)** | **2.77(1.29;4.26)** | **2.18(1.12;3.25)** | **2.00(0.95;3.04)** |
| Lipid disturbance |  |  |  |  |  |  |  |  |  |
| Poor(yes) | 0.00 (ref) | 0.00 (ref) | 0.00 (ref) | 0.00 (ref) | 0.00 (ref) | 0.00 (ref) | 0.00 (ref) | 0.00 (ref) | 0.00 (ref) |
| Ideal (no) | **1.56(0.59; 2.54)** | **1.45(0.69; 2.21)** | **1.22(0.59; 1.86)** | **2.47(1.50;3.44)** | **2.59(1.61;3.58)** | **2.63(1.75;.3.51)** | 0.85(-0.10;1.80) | 0.41(-0.52;1.34) | 0.44(-0.44;1.32) |
| High blood pressure |  |  |  |  |  |  |  |  |  |
| Poor (yes) | 0.00 (ref) | 0.00 (ref) | 0.00 (ref) | 0.00 (ref) | 0.00 (ref) | 0.00 (ref) | 0.00 (ref) | 0.00 (ref) | 0.00 (ref) |
| Ideal (no) | **2.37(1.83; 2.91)** | **1.98(1.54; 2.43)** | **1.74(1.31; 2.17)** | **3.59(2.91; 4.27)** | **3.51(2.87; 4.15)** | **3.24(2.64;3.84)** | **1.12(0.52;1.72)** | **0.95(0.42;1.48)** | **0.97(0.45;1.48)** |

Estimates were obtained by conducting a Laplace regression on the 25th, 50th, and 75th percentiles of age at death, adjusting for age at baseline, sex and educational level.

**Supplementary Table 8**. Results of the likelihood ratio tests building interaction terms considering age both as a continuous and as a categorical variable (< 65, >=65 years).

|  | **Age as continuous variable** | **Age as a categorical variable** |
| --- | --- | --- |
| **All causes death** | p= **0.0005** | p= **0.0018** |
| **CVD death** | p**<0.0001** | p**<0.0001** |
| **Non CVD death** | p=**0.0133** | p=0.1541 |

**Supplementary Table 9.** Hazard ratios (HRs) and 95% confidence interval (CIs) of death by the number of ideal cardiovascular health metrics by age.

|  | Ideal health metrics, n | *>=65 years at study entry* | | | | | *<65 years at study entry* | | | | |
| --- | --- | --- | --- | --- | --- | --- | --- | --- | --- | --- | --- |
|  |  | No. of cases | Person-years | Incidence rates (per1000) | HR (95% CI) | P-value for trend | No. of cases | Person-years | Incidence rates (per1000) | HR (95% CI) | P-value for trend |
| All causes |  |  |  |  |  |  |  |  |  |  |  |
|  | 0-1-2 | 163 | 4,116 | 37.81 | **1.94 (1.19-3.15)**^s,a^ | 0.003 ^s,a^ | 116 | 10,091 | 6.32 | **2.24 (1.37-3.66)** ^s,a^ | <0.0001 ^s,a^ |
|  | 3 | 402 | 12,009 | 33.87 | **1.43 (0.97-2.11)** ^s,a^ |  | 278 | 55,055 | 3.80 | **1.74 (1.21-2.49)** ^s,a^ |  |
|  | 4 | 734 | 24,778 | 30.29 | **1.65 (1.18 -2.31)** ^s,a^ |  | 518 | 134,463 | 3.56 | **1.67 (1.21 -2.31)** ^s,a^ |  |
|  | 5 | 744 | 26,108 | 28.49 | 1.32 (0.94-1.84) ^s,a^ |  | 359 | 161,556 | 2.47 | 1.19 (0.86-1.65) ^s,a^ |  |
|  | 6-7 | 322 | 12,686 | 25.58 | 1.00 (Ref) |  | 163 | 98,026 | 2.08 | 1.00 (Ref) |  |
| CVD death |  |  |  |  |  |  |  |  |  |  |  |
|  | 0-1-2 | 72 | 4,116 | 17.18 | **3.10 (1.36-7.08)** ^s,a^ | 0.009 ^s,a^ | 49 | 10,091 | 2.30 | **3.58 (1.30-9.86)** ^s,a^ | 0.001 ^s,a^ |
|  | 3 | 186 | 12,009 | 16.39 | **2.37 (1.22-4.59)** ^s,a^ |  | 60 | 55,055 | 0.79 | 1.49 (0.65-3.43) ^s,a^ |  |
|  | 4 | 298 | 24,777 | 12.34 | **2.29 (1.26-4.16)** ^s,a^ |  | 110 | 134,463 | 0.75 | 1.58 (0.75-3.32) ^s,a^ |  |
|  | 5 | 302 | 26,108 | 11.38 | **2.21 (1.22-4.00)** ^s,a^ |  | 48 | 161,556 | 0.34 | 0.64 (0.29-1.39) ^s,a^ |  |
|  | 6-7 | 104 | 12,686 | 8.50 | 1.00 (Ref) |  | 32 | 98,026 | 0.43 | 1.00 (Ref) |  |
| Non-CVD death |  |  |  |  |  |  |  |  |  |  |  |
|  | 0-1-2 | 91 | 4,116 | 20.63 | **1.43 (1.12-1.84)** ^e^ | 0.005 ^e^ | 67 | 10,091 | 4.02 | **2.39 (1.51-3.76)** ^a^ | <0.0001 ^a^ |
|  | 3 | 216 | 12,009 | 17.48 | 1.14 (0.94-1.38) ^e^ |  | 218 | 55,055 | 3.01 | **1.81 (1.32-2.48)** ^a^ |  |
|  | 4 | 436 | 24,778 | 17.95 | 1.11 (0.94-1.31) ^e^ |  | 408 | 134,463 | 2.81 | **1.71 (1.29-2.27)** ^a^ |  |
|  | 5 | 442 | 26,108 | 17.11 | 1.03 (0.88-1.22)^e^ |  | 311 | 161,556 | 2.13 | **1.36 (1.02-1.82)** ^a^ |  |
|  | 6-7 | 218 | 12,686 | 17.08 | 1.00 (Ref) |  | 131 | 98,026 | 1.65 | 1.00 (Ref) |  |

CVD: cardiovascular diseases. Incidence rates are standardized for sex and age (5-year band).

Cox proportional hazards models are adjusted for age, sex and educational level. Cox models were stratified for the covariates that did not satisfy the PH assumption (s,a: Stratified by sex and age; a: Stratified by age. e: Stratified by educational level).

**Supplementary Table 10**. Percentile Differences (PD) and 95% confidence interval (CIs) in age at death (years) according to the number of ideal cardiovascular health metrics.

|  |  | *>=65 years at study entry* | | | *<65 years at study entry* | | |
| --- | --- | --- | --- | --- | --- | --- | --- |
|  | Ideal health metrics, n | 25^th^ Percentile  PD (CI) | 50^th^ Percentile  PD (CI) | 75^th^ Percentile  PD (CI) | 25^th^ Percentile  PD (CI) | 50^th^ Percentile  PD (CI) | 75^th^ Percentile  PD (CI) |
| All causes |  |  |  |  |  |  |  |
|  | <=2 | 0.00 (ref) | 0.00 (ref) | 0.00 (ref) | 0.00 (ref) | 0.00 (ref) | 0.00 (ref) |
|  | 3 | 0.77(-0.69;2.24) | **1.53(0.37;2.70)** | 0.78(-0.74;2.31) | **2.83(1.45;4.21)** | **2.87(1.70;4.04)** | **2.89(1.69;4.09)** |
|  | 4 | **2.24(0.98;3.50)** | **2.07(1.03;3.10)** | 1.24(-0.27;2.74) | **3.64(2.41;4.87)** | **3.53(2.45;4.61)** | **3.55(2.44;4.66)** |
|  | 5 | **2.74(1.47;4.01)** | **2.57(1.54;3.60)** | **1.72(0.21;3.23)** | **5.65(4.36;6.94)** | **5.59(4.44;6.75)** | **5.61(4.43;6.79)** |
|  | 6-7 | **3.12(1.72;4.52)** | **3.26(2.14;4.38)** | **2.44(0.84; 4.04)** | **6.63(5.15;8.10)** | **6.58(5.23;7.94)** | **6.60(5.22; 7.98)** |
| CVD death |  |  |  |  |  |  |  |
|  | <=2 | 0.00 (ref) | 0.00 (ref) | 0.00 (ref) | 0.00 (ref) | 0.00 (ref) | 0.00 (ref) |
|  | 3 | 0.63(-0.99; 2.25) | 0.66(-0.80;2.12) | 0.17(-1.09; 1.43) | **7.39(4.65; 10.13)** | **6.78(4.43; 9.13)** | **6.75(4.42; 9.08)** |
|  | 4 | **1.98(0.57; 3.39)** | **1.82(0.42; 3.23)** | **1.51(0.28; 2.75)** | **7.83(5.33; 10.32)** | **7.23(5.14; 9.32)** | **7.20(5.13; 9.27)** |
|  | 5 | **2.51(1.08; 3.95)** | **2.35(0.95; 3.75)** | **2.21(0.95; 3.46)** | **12.79(9.94; 15.64)** | **12.23(9.70; 14.75)** | **12.17(9.67; 14.67)** |
|  | 6-7 | **3.81(2.20; 5.42)** | **4.00(2.45; 5.55)** | **3.64(2.21; 5.07)** | **11.14(8.00; 14.28)** | **10.58(7.71; 13.44)** | **10.53(7.68; 13.37)** |
| Non-CVD death |  |  |  |  |  |  |  |
|  | <=2 | 0.00 (ref) | 0.00 (ref) | 0.00 (ref) | 0.00 (ref) | 0.00 (ref) | 0.00 (ref) |
|  | 3 | 1.23(-0.58; 3.03) | 1.16(-0.28;2.59) | 0.88(-0.26; 2.03) | 1.06(-0.43; 2.55) | 1.41(-0.19;3.01) | 1.41(-0.19; 3.01) |
|  | 4 | **1.68(0.12;3.24)** | 1.22(-0.13;2.56) | **1.06(0.04; 2.08)** | **1.80(0.40;3.21)** | **2.15(0.64;3.66)** | **2.14(0.63; 3.65)** |
|  | 5 | **1.89(0.30;3.48)** | **1.66(0.31;3.00)** | **1.38(0.33; 2.42)** | **3.33(1.87;4.80)** | **3.68(2.11;5.24)** | **3.67(2.11; 5.23)** |
|  | 6-7 | **2.25(0.46;4.05)** | **1.71(0.26;3.16)** | **1.58(0.46; 2.70)** | **4.80(3.14;6.47)** | **5.15(3.40;6.91)** | **5.14(3.39; 6.90)** |

Estimates were obtained by conducting a Laplace regression on the 25th, 50th, and 75th percentiles of age at death, with number of ideal cardiovascular health metrics used as the main exposure and adjustment for age at baseline, sex and educational level.
